# New Insights Into Osteoclast Biology

**DOI:** 10.1002/jbm4.10539

**Published:** 2021-08-30

**Authors:** Michelle Maree McDonald, Albert Sungsoo Kim, Bridie S Mulholland, Martina Rauner

**Affiliations:** ^1^ Bone Biology Program, Healthy Ageing Theme Garvan Institute of Medical Research Sydney NSW Australia; ^2^ St Vincent's Clinical School Faculty of Medicine UNSW Sydney Sydney NSW Australia; ^3^ Department of Diabetes and Endocrinology Royal North Shore Hospital St Leonards NSW Australia; ^4^ Department of Diabetes and Endocrinology Westmead Hospital Westmead NSW Australia; ^5^ School of Pharmacy and Medical Sciences Griffith University Gold Coast QLD Australia; ^6^ Menzies Health Institute Queensland Griffith University Gold Coast QLD Australia; ^7^ Department of Medicine III Medical Faculty of the Technische Universität Dresden Dresden Germany; ^8^ Center for Healthy Aging Medical Faculty of the Technische Universität Dresden Dresden Germany

**Keywords:** THERAPEUTICS, ANTIRESORPTIVES, DISEASES AND DISORDERS OF/RELATED TO BONE, OSTEOPOROSIS, DISEASES AND DISORDERS OF/RELATED TO BONE, OSTEOPETROSIS, CELLS OF BONE, OSTEOCLASTS, BONE MODELING AND REMODELING, MOLECULAR PATHWAYS ‐ REMODELING

## Abstract

Osteoclasts are multinucleated cells that are characterized by their unique ability to resorb large quantities of bone. Therefore, they are frequently the target of therapeutic interventions to ameliorate bone loss. In an adult organism, osteoclasts derive from hematopoietic stem cells and differentiate into osteoclasts within a multistep process under the influence of macrophage colony‐stimulating factor (M‐CSF) and receptor activator of NF‐κB ligand (RANKL). Historically, the osteoclast life cycle has been defined as linear, whereby lineage‐committed mononuclear precursors fuse to generate multinucleated highly specialized and localized bone phagocytic cells, which then undergo apoptosis within weeks. Recent advances through lineage tracing, single cell RNA sequencing, parabiosis, and intravital imaging approaches have challenged this dogma, revealing they have greater longevity and the capacity to circulate and undergo cell recycling. Indeed, these new insights highlight that under homeostatic conditions very few incidences of osteoclast apoptosis occur. More importantly, as we revisit the formation and fate of the osteoclast, novel methods to target osteoclast biology in bone pathology and regeneration are emerging. This review briefly summarizes the historical life cycle of osteoclasts and highlights recent discoveries made through advanced methodologies, which have led to a paradigm shift in osteoclast biology. These findings are discussed in light of both existing and emerging bone targeted therapeutics, bone pathologies, and communication between osteoclasts and cells resident in bone or at distant sites. © 2021 The Authors. *JBMR Plus* published by Wiley Periodicals LLC on behalf of American Society for Bone and Mineral Research.

## Introduction

Bone and mineral homeostasis is critically dependent on the balance of bone resorption via osteoclasts and bone formation via osteoblasts. However, in several bone diseases, bone remodeling is out of balance, resulting in bone loss. In fact, excessive osteoclastic bone resorption causes bone loss in the most prevalent forms of osteoporosis, including postmenopausal osteoporosis as well as bone loss due to inflammation and malignancy. Thus, the osteoclast has been a central target for many anti‐osteoporosis therapies, showing efficacious results in halting bone loss and preventing fractures in various pathological conditions. Recent clinical research, however, has highlighted new phenomena occurring during or after osteoporosis treatment, such as the “rebound effect” after discontinuation of denosumab,^(^ ^1^ ^)^ suggesting that the biology of the osteoclast is not fully understood. A more comprehensive understanding, therefore, is required to optimize existing treatments and develop new ones.

Osteoclasts are the primary bone resorbing cells.^(^ ^2^ ^)^They are highly specific multinucleated phagocytic cells of hematopoietic origin and are characterized by distinct features such as the ruffled border, which facilitates demineralization and degradation of bone matrix.^(^ ^2^ ^)^ After having established for some time that osteoblasts in co‐culture can promote osteoclast formation,^(^ ^3^ ^)^ the discovery of receptor activator of NF‐κB ligand (RANKL) as that crucial factor and key differentiation factor for osteoclasts greatly facilitated knowledge on osteoclast biology in the early 1990s and eventually led to the development of a RANKL‐neutralizing antibody, denosumab, which today is one of the most potent therapeutics to block osteoclasts and bone resorption and decrease fracture risk in patients with osteoporosis.^(^ ^4^ ^)^ Despite decades of research into their origin, formation, function, and fate, new insights into these unique cells continue to emerge adding more insights into their communication with other bone‐residing cells, their capacity to repopulate from circulating cells and even recycle themselves, all in order to maintain a previously unappreciated longevity and avoid cell death. It is through these discoveries that the fundamentals of osteoclast biology are being reworked, ultimately leading to an improved understanding of bone pathologies and optimal treatment strategies for the millions of patients affected by osteoclast‐related bone diseases.

This opinion‐based narrative review summarizes novel insights into osteoclasts from recent years, focusing on their origin, formation, and fate, as well as their communication with osteoblasts. Moreover, the importance of these new concepts for current osteoporosis treatments and the identification of new targets is discussed.

## Osteoclast Origin and Formation

### Origin

**Fig 1:**
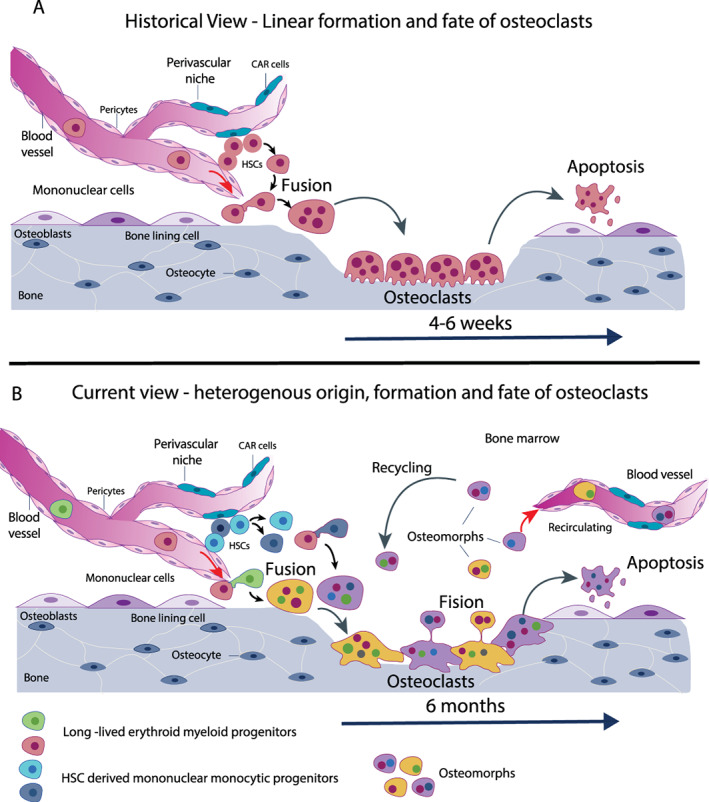
Historic and current view of osteoclastogenesis. (*A*) Historical view: osteoclasts develop from hematopoietic stem cell‐derived monocyte precursors, fuse, and eventually form mature osteoclasts sitting on the bone surface. After bone resorption, osteoclasts undergo apoptosis within 4 to 6 weeks. (*B*) Current view: osteoclasts form through iterative fusion of HSC–derived monocyte precursors (blue and dark blue) partially with syncytia that originated from the erythroid‐myeloid precursors during embryogenesis (green and red). Throughout bone resorption, mature osteoclasts undergo fission into osteomorphs (red, green, and blue). These either enter into the circulation or are recycled and fuse with other osteoclast syncytia to form new osteoclasts or undergo apoptosis. Osteoclasts in mice survive up to 6 months. HSC = hematopoietic stem cell.

Since the beginning of the last century, researchers have been keen on identifying the origin of osteoclasts, with the result of several hypotheses being proposed in the early 1900s; eg, that osteoclasts from regenerating salamander limbs derive from fusion of mesenchymal cells,^(^ ^5^ ^)^ osteoblasts,^(^ ^6^ ^)^ liberated chondrocytes,^(^ ^7^ ^)^ lymphocytes,^(^ ^8^ ^)^ or monocytes.^(^ ^9^, ^10^ ^)^ These studies were based on autoradiographs taken from tissues after various time points of a pulse label for tritiated [^3^H]‐thymidine to label proliferating cells. However, because [^3^H]‐thymidine labels all proliferating cells, these studies could not clearly discriminate between cell types or the origin of the labeled cell; namely, whether it derived from the local tissue or whether it invaded that tissue via the vascular system. A subsequent study by Kahn and Simmons^(^ ^11^ ^)^ resolved this issue using a chimeric system composed of limbs and bone rudiments from the Japanese quail grown in the chorioallantoic membrane of the chicken embryo. Using the distinct appearance of the interphase nuclei of the quail versus the chick and the vascularization and blood supply of the grafts from the host, the authors concluded that most osteoclasts derived from mononucleated hematogenous cells such as monocytes and that some osteoclasts derived from fusion of bone cells in situ. This study was later supported by parabiosis experiments and bone marrow transplantation studies in osteopetrotic animals which unequivocally showed that osteoclasts in adult animals derive from circulating mononuclear precursors of the hematopoietic system (Figure 1A).^(^ ^12^, ^13^, ^14^ ^)^

Today, more refined lineage tracing and fate‐mapping experiments, detailed characterization of time‐course parabiosis experiments, and genetic deletion of key factors required for osteoclastogenesis during distinct stages of development provide new fundamental insights into the origin of osteoclasts and their lifespan.^(^ ^15^, ^16^ ^)^ During embryonic development and the neonatal stage, erythroid‐myeloid progenitors (EMPs), which appear at around embryonic day 7 in the blood island of the yolk sac and can differentiate into colony stimulating factor 1 receptor (Csf1r)+ yolk sac macrophages, serve as precursors for osteoclasts that can create space for postnatal bone marrow hematopoiesis. In postnatal life, it has been suggested that these precursors are gradually replaced by hematopoietic stem cell (HSC)‐derived mononuclear monocytic precursor cells that fuse, in part also with long‐lived EMP‐derived osteoclasts, to form and maintain osteoclasts throughout life.^(^ ^15^ ^)^ Detailed calculations based on time‐course parabiosis experiments are indicative that circulating monocytic precursor cells fuse with existing osteoclasts one at a time about every 4 to 8 weeks, that a mouse osteoclast has on average five nuclei, and that individual nuclei in osteoclast syncytia are replaced about every 2 months.^(^ ^15^ ^)^ Under steady state, only about 0.5% to 2% of osteoclasts at a given time acquire a new precursor cell, suggesting that osteoclast turnover is rather slow.^(^ ^15^ ^)^ Osteoclast fusion processes, however, can be markedly accelerated after treatment with RANKL, a key differentiation factor for osteoclasts,^(^ ^17^ ^)^ or in states of injury and inflammation.^(^ ^18^ ^)^ The concept that osteoclasts generate through iterative fusion of monocytic precursors is supported by data showing that the phenotype of osteopetrotic mice (ie, for instance lack of tooth eruption and the absence of a bone marrow cavity) can be rescued by transfusion of neonatal osteopetrotic mice with monocytes.^(^ ^15^ ^)^ These findings may have therapeutic implications suggesting that congenital osteopetrosis could not only be treated with bone marrow transplantation, but via blood transfusions, which is a less harsh procedure compared to bone marrow transplantation. In addition, these findings propose a relatively long lifespan of osteoclasts. Using 5‐ethynyl‐2′‐deoxyuridine (EdU) labeling as well as pulse‐chase and cell‐fate experiments, two independent groups showed that EMP‐derived osteoclasts were still alive 6 months after birth.^(^ ^15^, ^16^ ^)^ These long‐lived cells were shown to contribute to osteoclastogenesis not only in steady state, but also during fracture healing in adult mice. Thus, as opposed to previous literature suggesting a rather limited lifespan of osteoclasts of a few days up to 6 weeks,^(^ ^19^, ^20^, ^21^, ^22^ ^)^ these data suggest that some osteoclasts which were generated during embryonic development still prevail during adulthood, likely through replenishment through iterative fusion with new precursor cells in the circulation (Figure 1B). Because these studies were limited to an observation period of 6 months, it is uncertain whether these osteoclast syncytia potentially remain alive throughout the entire lifespan. Also, it is unclear whether osteoclast syncytia, derived from different ratios of EMP‐derived versus HSC‐derived precursors, have the same resorptive capacity. Nonetheless, these developments in osteoclast biology potentially provide exciting new avenues to address bone resorption pathologies; however, they require further studies and validation.

Osteoclasts require two cytokines that are essential for their formation: macrophage colony‐stimulating factor (M‐CSF) and RANKL.^(^ ^2^, ^23^, ^24^, ^25^ ^)^ Although M‐CSF is required for the differentiation of HSC into the monocyte/macrophage lineage, promotes the proliferation, and extends the lifespan of these precursor cells,^(^ ^26^, ^27^ ^)^ RANKL is important for osteoclast differentiation, fusion, and lifespan of mature osteoclasts.^(^ ^2^ ^)^ Global deficiency of these cytokines or their receptors (M‐CSFR [*Csfr1r*] and RANK [*Tnfrsf11a*]) results in the absence of osteoclasts and thus, an osteopetrotic phenotype with the typical characteristics of marble bone, lack of tooth eruption, and lack of a functional bone marrow cavity, resulting in extramedullary hematopoiesis.^(^ ^23^, ^28^ ^)^ Knockout of M‐CSFR or RANK in EMPs also results in an early osteopetrotic phenotype that, however, resolves over time once HSC‐derived osteoclastogenesis occurs during postnatal life.^(^ ^15^ ^)^ On the other hand, deficiency of these receptors in HSCs leads to normal bone development, but after 4 to 5 months, mice start to develop high bone mass due to a deficiency of osteoclasts, again indicating that both embryonically EMP‐derived and HSC‐derived osteoclasts are likely essential to maintain osteoclasts throughout life.^(^ ^15^ ^)^

Although the HSC and its myeloid descendants clearly provide the precursor cells for osteoclasts, to date there are controversies as to whether only monocytes/macrophages or also dendritic cells can provide precursor cells for osteoclasts. Both cell types derive from HSC via the common myeloid progenitors down to the granulocyte colony‐stimulating factor (G‐CSF)/M‐CSF progenitors and thus, a certain degree of plasticity may occur between these cell types. Several studies have shown that immature CD11c+ dendritic cells have the ability to differentiate into osteoclasts in vitro, resorb bone, and have an equivalent expression of main osteoclast markers as monocyte‐derived osteoclasts, especially during pathological (inflammatory) conditions.^(^ ^29^, ^30^, ^31^, ^32^ ^)^ Moreover, transfer of immature CD11c+ cells into osteopetrotic mice led to osteoclast formation and ameliorated the bone phenotype.^(^ ^29^, ^32^ ^)^ On the other hand, mice lacking mature dendritic cells do not show alterations in osteoclast numbers.^(^ ^33^ ^)^ Single‐cell RNA sequencing of in vitro differentiated osteoclasts from bone marrow cells has provided more detailed insights into the various stages of osteoclast lineage commitment. The most immature committed monocytic precursor that is characterized by high expression of M‐CSFR, RANK, C‐X3‐C motif chemokine receptor 1 (CX3CR1), C–C motif chemokine ligand 2 (CCL2), and MAF BZIP transcription factor b (MAFB) undergoes stepwise differentiation spanning four intermediate populations until the final mature osteoclast population with a high expression of cathepsin K, tartrate‐resistant‐acid phosphatase 5, matrix metalloproteinase 9, and dendritic cell‐specific transmembrane protein (DC‐STAMP) arises.^(^ ^34^ ^)^ Within the second population, cells show a dendritic cell–like expression pattern with a transient expression of cluster of differentiation 11c (CD11c). Deficiency of RANK in CD11c+ cells inhibited osteoclast formation in vitro and in vivo, indicating that this CD11c+ dendritic cell‐like precursor population is important for proper osteoclastogenesis. Further, using RNA expression in single cells, CREB‐binding protein/p300‐interacting transactivator with Glu/Asp‐rich carboxy‐terminal domain 2 (CITED2) was identified as a novel critical factor driving terminal differentiation of osteoclasts, which previously has not been identified in bulk sequencing approaches.^(^ ^34^ ^)^ Given the limitations of extracting osteoclasts from in vivo samples, these single‐cell RNA outcomes are driven from in vitro differentiated osteoclasts derived from primary murine bone marrow; hence, assumptions are made that these findings are relevant in vivo and in humans. Despite this limitation, however, considering the high plasticity of not only monocytes, macrophages, and dendritic cells, but also virtually all cell types that are now arising from single‐cell RNA sequencing data sets, it can be expected that several markers will be soon identified that more accurately characterize specific osteoclast (sub)‐populations.

### Communication of osteoclasts with osteoblast‐lineage cells

Stromal cells, osteoblasts, and osteocytes in the bone microenvironment are critical regulators of osteoclast differentiation because they are the main producers of M‐CSF and RANKL,^(^ ^3^, ^35^, ^36^ ^)^ although immune cells can also induce their expression during pathological conditions such as hormone deficiency or inflammation.^(^ ^37^, ^38^ ^)^ The discovery that stromal cells are an important source of cytokines necessary to induce osteoclast differentiation was pivotal for the emergence of the concept of “coupling”, even before M‐CSF and RANKL were identified as those critical factors.^(^ ^3^, ^39^ ^)^ Coupling refers to the coordinated actions of osteoclasts and osteoblasts and their intimate communication with each other.^(^ ^40^ ^)^ As such, genetic or therapeutic suppression of osteoclasts leads to a reduction in osteoblast activity and bone formation as well (eg, treatment with bisphosphonates or denosumab), whereas stimulation of osteoblasts results in enhanced osteoclast activity. The best example of therapeutics impacting coupling between osteoblast and osteoclasts is that exhibited when parathyroid hormone (PTH) is targeted. PTH, a well‐established regulator of bone homeostasis and exogenous delivery of PTH—dependent on the manner in which it is delivered—has been shown to alter both osteoblast and osteoclast activity. Stable levels of PTH form a physiological component of calcium homeostasis, whereby PTH is secreted in a continuous fashion to stimulate RANKL‐induced osteoclast formation; conversely, intermittent peaks of PTH, such as seen in PTH treatment of osteoporosis, exerts a potent anabolic effect that relies on both bone formation and bone resorption.^(^ ^41^, ^42^ ^)^

Extensive work by the Partridge laboratory has shown that monocyte chemoattractant protein 1 (MCP‐1) is a pivotal coupling factor to both the catabolic and anabolic action of PTH and, thus, has identified MCP‐1 as a key osteoblast‐derived regulator of osteoclast activity.^(^ ^43^, ^44^, ^45^ ^)^ MCP‐1 is a member of the C‐C chemokine family as CCL2 and is a crucial regulator of osteoclast biology.^(^ ^41^ ^)^ Early studies of the relationship between MCP‐1 and osteoclasts identified the chemokine as a coupling factor—MCP‐1 is expressed on osteoblasts and binds its receptor, C‐C motif chemokine receptor 2 (CCR2), on osteoclast precursors to drive osteoclast differentiation.^(^ ^46^, ^47^ ^)^ PTH treatment, irrespective of whether it is continuous or intermittent, induces osteoblastic expression of MCP‐1 to recruit osteoclast precursors. Notably, Siddiqui and colleagues^(^ ^44^ ^)^ showed a deficiency of fully functioning osteoclasts in MCP‐1−/− mice following continuous infusion of PTH and in the presence of normally induced RANKL expression that was sufficient for forming functioning osteoclasts in control mice. More recently, the same group showed that intermittent PTH treatment enhances transforming growth factor‐β (TGF‐β) signaling, and that this enhancement is lost in the absence of MCP‐1, implicating TGF‐β signaling in the action of intermittent PTH.^(^ ^45^ ^)^ The use of seven‐amino acid truncated (7ND), a dominant negative form of MCP‐1, to inhibit the action of the chemokine has highlighted the importance of this osteoblast‐induced coupling factor to osteoclast formation.^(^ ^48^, ^49^, ^50^ ^)^ As such, 7ND has gained attraction as an effective MCP‐1‐inhibitory agent, identifying itself as a novel therapeutic and MCP‐1 as a novel treatment target in a range of diseases.^(^ ^51^, ^52^ ^)^

Finally, a long‐standing central question of the past that was recently resolved was whether the membrane‐bound or the soluble membrane‐shed form of RANKL is required for osteoclastogenesis and, hence, whether direct cell–cell contact is necessary to induce osteoclastogenesis or whether RANKL secretion by stromal cells is sufficient. Despite several insights from direct and indirect co‐cultures, conclusive evidence in vivo was still lacking. Using transgenic mice with a sheddase‐resistant form of RANKL, Xiong and colleagues^(^ ^53^ ^)^ showed that membrane‐bound RANKL is required for optimal skeletal development and responsible for pathological bone loss, but that soluble RANKL also contributes to bone remodeling during steady state in adult mice. Although robust and convincing data for the roles of both forms of RANKL in bone homeostasis exist, whether or not these outcomes reflect the human setting, however, remains to be clarified. With the emergence of intravital imaging in bone, mounting evidence for direct osteoclast–osteoblast interactions exists.^(^ ^54^, ^55^ ^)^ Indeed, this approach revealed a novel serine protease inhibitor, secretory leukocyte protease inhibitor (SLPI), as a key regulator of PTH‐mediated bone anabolism through direct osteoblast–osteoclast interactions^(^ ^56^ ^)^ and is likely to unveil further key coupling factors.

## Osteoclast Maintenance, Function, and Fate—Historical and Advances through Imaging

### Maintenance and function

Upon maturation and adhesion to bone surfaces, osteoclasts function through the formation of a sealing zone rich in F‐actin to form a ruffled border at the membrane designed to release protons and proteases to demineralize and break down bone matrix. Upon completion of this function they are typically thought to undergo apoptosis at the end of their life cycle.^(^ ^57^ ^)^ Numerous survival factors pivotal to both osteoclast differentiation and maintenance are produced by but not limited to bone resident stromal cells, osteoblasts, and osteocytes. These include soluble and membrane‐bound RANKL,^(^ ^2^, ^53^, ^58^ ^)^ M‐CSF,^(^ ^59^ ^)^ MCP‐1,^(^ ^45^, ^46^, ^48^ ^)^ as described above in the communication of osteoclasts and osteoblasts section, and secreted cytokines such as tumor necrosis factor α (TNF‐α)^(^ ^60^ ^)^ and interleukin 1 (IL‐1).^(^ ^61^ ^)^ Importantly local factors such as matrix ligands, eg, vitronectin, are essential to osteoclast adhesion through alpha(v)beta3 integrins.^(^ ^62^ ^)^ Further, matrix collagen^(^ ^63^ ^)^ and extracellular acidosis^(^ ^64^ ^)^ are also important factors driving fusion and resorption by osteoclasts at the bone surface. Although not critical to osteoclast survival, the capacity for osteoclasts to resorb bone via their specialized ruffled border is highly dependent on the process of membrane trafficking.^(^ ^65^ ^)^ Recent work has highlighted multiple novel osteoclast‐specific therapeutic targets, which will inhibit their function rather than formation or survival, thereby negating possible feedback mechanisms such as those driving denosumab rebound bone loss, as discussed later in the osteoclast targeted thearpeutics section.

Recent advances in imaging using intravital microscopy have accelerated our understanding of osteoclast function and revealed novel heterogeneity within the osteoclast population in vivo through dynamic imaging of mature osteoclasts within their native and complex environment. The use of pH‐sensitive fluorescent probes and fluorescent proton pump reporter mice allow localization of protons pumps and acid release to the sealing zone of active osteoclasts *in vivo* within the calvaria.^(^ ^66^, ^67^ ^)^ Further, quantification of resorbing activity was obtained in real time, revealing that osteoclasts that were non‐motile were acidifying and therefore resorbing bone, whereas more motile osteoclasts were showing less acidification and therefore reduced bone resorptive capacity. The impact of different nitrogen‐containing bisphosphonates on osteoclast function and motility was also assessed using this imaging approach, revealing not only reductions in acidification within 12 hours of treatment, but also increases in osteoclast motility.^(^ ^68^ ^)^ It would be of interest to examine the effect of non‐nitrogen–containing bisphosphonates such as clodronate using this approach because these agents are more likely to induce osteoclast apoptosis. To date these approaches to dynamically image osteoclasts in vivo are limited to mice; therefore, the relevance of these outcomes to human osteoclasts requires clarification. In addition to developments in our understanding of osteoclast function, three‐dimensional (3D) *in vivo* static and dynamic imaging in bone has revealed morphological characteristics that standard two‐dimensional (2D) histological assessment overlooks. These include the fact that these cells are of a stellate structure and form a syncytium on the bone surface,^(^ ^17^, ^55^ ^)^ allowing us to revisit interpretations from serial 2D static sections made many decades ago.^(^ ^69^ ^)^

Osteoclasts have high energy demands during formation and resorption; therefore, mitochondrial function is pivotal to their survival and function. Despite this, osteoclast metabolism is poorly understood. This is likely because of the multistep processes during osteoclast differentiation and maturation, each step in this complex process requiring complex metabolic adjustments.^(^ ^70^ ^)^ Nevertheless, in vitro glycolysis, oxidative phosphorylation, and lactate production increase during RANKL‐induced osteoclast differentiation.^(^ ^71^ ^)^ This increase in oxidative phosphorylation as osteoclasts fuse and mature was further associated with increases in size and abundance of mitochondria. Glucose metabolism is accelerated during osteoclast differentiation as the expression of glucose transporter 1 (GLUT1) increases,^(^ ^72^ ^)^ and a metabolic shift toward mitochondrial respiration enhances osteoclast differentiation.^(^ ^73^ ^)^ In vivo, the disruption of mitochondrial complex 1 led to an osteopetrotic phenotype with impaired osteoclast formation and function.^(^ ^74^ ^)^ However, bone resorption by mature osteoclasts was shown to be more dependent on active aerobic glycolysis than oxidative phosphorylation,^(^ ^70^ ^)^ with pharmacological inhibition of glycolysis blocking bone resorption in vivo.^(^ ^75^ ^)^ Mitochondria, as well as a source of adenosine triphosphate (ATP), are a reservoir for pro–cell death proteins such as cytochrome c.^(^ ^76^ ^)^ Hence, one could hypothesize that the release of these mitochondrial pro‐apoptotic factors once an osteoclast reaches a threshold of size may be a mechanism for controlling not only osteoclast function but also fate.

### Osteoclast fate and recycling

A number of endogenous factors are suggested to drive osteoclast apoptosis^(^ ^77^ ^)^; these include elevated extracellular calcium,^(^ ^78^ ^)^ estrogen,^(^ ^79^, ^80^ ^)^ TGF‐β1,^(^ ^81^ ^)^ semaphorin‐3A (sema3A),^(^ ^82^ ^)^ and osteoprotegerin (OPG).^(^ ^83^, ^84^ ^)^ Many of these factors work through inhibition of RANKL signaling; therefore, they could simply be opposing pro‐osteoclastic factors, rather than driving apoptosis per se. In addition, conflicting data exist for many of these so‐called pro‐apoptotic factors. OPG has been shown to suppress osteoclast apoptosis through inhibiting TNF‐related apoptosis‐inducing ligand (TRAIL),^(^ ^85^ ^)^ and TGF‐β signaling has been shown to induce pro‐osteoclast survival factors leukemia inhibitory factor (LIF) and suppressor of cytokine signaling 3 (SOCS3).^(^ ^86^ ^)^ Further, estrogen therapy was shown to decrease the number of osteoclasts on bone surfaces, but increase osteoclasts within the marrow space away from bone.^(^ ^87^ ^)^ Rather than osteoclast apoptosis as a mechanism, this may be interpreted as changes in osteoclast morphology or a suppression in osteoclast formation and accumulation of precursor cells.

Technological advances such as small conditional RNA (scRNA) sequencing and intravital imaging have facilitated revelations not only in osteoclast function but also in osteoclast biology. Recently, these approaches were combined to define a new cell in the osteoclast lineage, the osteomorph (Fig. 1B).^(^ ^17^ ^)^ In the absence of a clean single‐osteoclast reporter, the authors exploited established osteoclast biology to track the fate of osteoclasts in vivo. Using a mixed bone marrow chimera system in which osteoclasts formed through the fusion of two mononuclear cells, each carrying a different fluorescent reporter driven by an osteoclast gene (LysozymeM‐tdTomato and CSF1R or Blimp‐1‐GFP), leading to multinucleated osteoclasts carrying both Tdtomato and GFP expression. In addition, the presence of a fluorescent bisphosphonate within the cells confirmed their role in prior active resorption. Dynamic imaging of these cells under the influence of RANKL revealed that osteoclasts undergo fusion and fission in vivo.^(^ ^17^ ^)^ This is the first clear evidence that osteoclasts can undergo fission in vivo, and interestingly supports inferences from static imaging that were made over four decades ago.^(^ ^88^ ^)^ Of note, the concept of osteoclast fission had been described in vitro.^(^ ^89^ ^)^ The fission products were shown to re‐fuse with other osteoclasts, a process referred to as osteoclast recycling. The fission products, osteomorphs, were detected in blood and upon isolation from bone marrow were defined as a unique cell population expressing 151 unique genes when compared to osteoclasts and osteoclast precursors using scRNA sequencing (scRNAseq). Within this osteomorph signature, a number of novel cell surface protein coding genes including AXL, CCR3, VCAM1, CD74, and CAM1 were confirmed to be upregulated. Further, 17 genes upregulated in osteomorphs were associated with bone structural or functional phenotypes. OPG:Fc treatment led to the ablation of osteoclasts and an accumulation of osteoclast precursors and osteomorphs. Taken together, it was concluded that osteoclast recycling through osteomorphs is an alternate cell fate to apoptosis, providing a pool of primed osteoclast precursors, which can re‐fuse to form active osteoclasts under specific direction. This phenomenon also supports the extended cell lifespan and circulation of osteoclasts and precursors as described by Jacome‐Galarza and colleagues.^(^ ^15^ ^)^

One could also hypothesize alternative roles for these fission products or osteomorphs in maintaining bone homeostasis. First, because fission products were shown to be capable of bone resorption in vitro (PY Ng and NJ Pavlos, unpublished data, 2013), in addition to circulating and migrating within bone, they could remain on bone surfaces and perform more shallow pit resorption rather than more aggressive trench resorption, as described by Soe and Delaisse.^(^ ^90^ ^)^ One could hypothesize that a pit‐forming osteoclast, which is resorbing less bone for shorter periods, would require reduced metabolic processes, reduced mitochondrial activity, and potentially less nuclei than an osteoclast driving aggressive trench bone resorption. Indeed, this is supported by recent in vitro analyses of human osteoclasts, showing trench‐forming osteoclasts had higher numbers of nuclei.^(^ ^91^ ^)^ Hence, osteoclast fission products remaining on bone could drive less aggressive bone resorption, with reduced acidification and could indeed exhibit higher motility, as discussed above in the osteoclast maintenance and function section. Second, osteoclast fission products remaining on bone could contribute to secondary resorption that takes place during the reversal phase behind primary resorption and in doing so coordinate reversal phase osteogenesis as described by Lassen and colleagues.^(^ ^92^ ^)^ Although not closely examined by these authors, secondary osteoclasts could also be smaller with less nuclei and perform resorptive processes while also coordinating subsequent bone formation. Indeed, this would suggest that coupling between bone resorption and formation via osteoclast‐driven factors is another potential role for osteomorphs in maintaining bone homeostasis. Of note is the fact that osteoclast fission has only been reported by these authors and so far the data is limited to murine osteoclasts. Confirmation of this finding through additional murine studies, and demonstration that human osteoclasts undergo a similar fate, is necessary.

As mentioned previously in the communication of osteoclasts with osteoblasts section, osteoblast lineage cells regulate osteoclast formation through the production of coupling factors such as RANKL and MCP‐1. The production of pro‐osteoblastic factors by osteoclasts, on the other hand, has also been well established and includes the release of growth factors from the bone matrix through resorption such as TGF‐β and insulin‐like growth factor 1 (IGF‐1),^(^ ^93^ ^)^ the production of cytokines such as LIF^(^ ^94^ ^)^ and cardiotrophin‐1 (CT‐1),^(^ ^95^ ^)^ and secretion of Wnt inhibitory factor‐1 (WIF1).^(^ ^96^ ^)^ The release of vesicular RANK by osteoclasts during maturation stimulates RANKL reverse signaling and downstream Runt‐related transcription factor 2 (runx2)‐driven osteogenesis in osteoblasts.^(^ ^97^ ^)^ Conversely, osteoclasts are known to suppress osteoblast formation through expression of Semaphorin 4D^(^ ^93^ ^)^ and sclerostin.^(^ ^96^ ^)^ Recently, using single cell sequencing approaches in human tissue samples, dipeptidylpeptidase‐4 (DPP4) was defined as an osteoclast‐derived coupling factor, which was also shown to improve glycemic control in diabetic patients when its systemic secretion was reduced during denosumab treatment.^(^ ^94^ ^)^ Coupling between osteoclasts and osteoblasts can also occur through direct cell contact. Intravital imaging of these cell populations in the calvaria revealed distinct regions where osteoclasts that had direct contact with osteoblasts had reduced resorptive function.^(^ ^55^ ^)^ Interestingly, intermittent PTH treatment for 3 or 6 weeks increased the number of osteoclast–osteoblast direct cell contacts, which may suggest the presence of more membrane‐bound osteoblast–osteoclast coupling factors than previously appreciated. It would be of interest to examine osteomorph dynamics and transcript signature in light of osteoclast–osteoblast coupling dynamics. Despite these extensive studies, our ability to therapeutically “un‐couple” osteoclast–osteoblast interactions to improve bone mass is limited to PTH. PTH is currently used in the clinic to build bone mass, but we are still discovering the mechanisms through which this is achieved, as mentioned previously in the communication of osteoclasts with osteoblasts section.^(^ ^56^ ^)^ Hence, further work is needed here to develop a therapy that specifically targets coupling. The potential clinical impact of an agent that can achieve increased bone formation and reduced resorption could be a significant step forward in this field.

## Osteoclast Targeted Therapeutics—Current and Future

The osteoclast has been the direct target of a number of therapeutics aimed to prevent bone loss across a spectrum of bone diseases. In particular, anti‐resorptive agents have targeted the formation of osteoclasts, or their functional capacity, achieving great success in the clinical management of osteoporosis and other low bone mass disorders. These include, bisphosphonates and denosumab. Bisphosphonates are inorganic compounds that bind to hydroxyapatite in the bone matrix and target osteoclasts through their uptake during active bone resorption. Upon ingestion, bisphosphonates drive either osteoclast apoptosis or disrupt cellular function via inhibition of the mevalonate pathway and downstream guanosine triphosphatase (GTPase) signaling.^(^ ^98^ ^)^ For decades, bisphosphonates have been the gold standard approach used in the clinical management of osteoporosis, cancer‐induced bone disease, and other bone pathologies with underlying increased bone resorption.

More recently, denosumab—a fully humanized monoclonal antibody that binds to RANKL—has become a frontline therapy for osteoporosis, with its long‐term, durable anti‐fracture efficacy shown in the Fracture Reduction Evaluation of Denosumab in Osteoporosis Every 6 Months (FREEDOM) trial and its extensions.^(^ ^4^, ^99^ ^)^ Denosumab inhibits RANKL‐RANK interaction, thereby preventing differentiation, maturation, and survival of osteoclasts and subsequent bone resorption.^(^ ^100^ ^)^ With initiation of denosumab therapy, there is a rapid reduction in biochemical markers of bone resorption, specifically the C‐terminal cross‐linked telopeptide of type 1 collagen and osteoclastic enzyme tartrate resistant acid phosphatase type 5.

Odanacatib is a small molecular inhibitor of the protease cathepsin K, which is required by osteoclasts to degrade organic bone matrix. Odanacatib had success in early clinical trials, showing a strong capacity to inhibit bone resorption without suppressing bone formation, thereby potently increasing bone mass and reducing fractures. However, during phase III clinical trials, Odanacatib treatment was associated with increased risk of cardiovascular events, in particular stroke, leading the developers to cease trials and stop pursing its development for clinical use.^(^ ^101^ ^)^ However, these trials did show that by targeting osteoclast function rather than formation, the crosstalk from osteoclasts to osteoblasts can be maintained with the benefit of leaving bone formation intact despite disruption of bone resorption.

Despite the success of anti‐resorptive therapies such as bisphosphonates and denosumab in preventing bone loss and fracture, thereby improving quality of life and survival, rare but clinically significant side effects of long‐term use of these agents can heavily impact life. Atypical femoral fractures are attributed to long‐term use of both bisphosphonates and denosumab and cause morbidity and often require costly surgical intervention.^(^ ^102^, ^103^ ^)^ It is hypothesized that they occur due to reduced turnover of cortical bone and therefore increased brittleness and accumulation of microdamage, leading to structural weakness.^(^ ^104^ ^)^ In addition, the complication of osteonecrosis of the jaw is attributed to long‐term anti‐resorptive use.^(^ ^105^ ^)^ As a result of these complications, drug holidays are often implemented. Due to the binding capacity and long‐term residual buildup of bisphosphonates in bone, interruption of treatment maintains protection from fractures.^(^ ^106^ ^)^ However, following denosumab discontinuation, clearance of circulating antibodies leads to rapid offset of treatment effect, driving the rapid rise in bone turnover markers to levels above pretreatment levels.^(^ ^99^ ^)^ Clinically, this manifests as rebound bone mineral density loss across all skeletal sites and particularly in the lumbar spine, which correlates with the rise in bone turnover markers and development of spontaneous vertebral fractures.^(^ ^1^ ^)^ Evidence in murine models that upon withdrawal of OPG:Fc—the endogenous RANKL decoy receptor denosumab mimics—accumulated osteomorphs and osteoclast precursors rapidly re‐fused to form osteoclasts which actively resorbed bone; this provides a potential mechanism for this rebound reduction in bone mass.^(^ ^17^ ^)^ This body of work therefore not only highlights our emerging capacity for discoveries in bone cell biology, but the ability to define mechanisms behind bone therapeutic challenges. Numerous studies have explored sequential therapy with bisphosphonates following denosumab cessation, aiming to prevent bone loss. However, results are varied, and an optimal dosing strategy has not been defined yet.^(^ ^107^ ^)^

Mechanistically, serum RANKL levels rise following denosumab discontinuation, though this only reached statistically significant levels after 12 months following loss of denosumab effect.^(^ ^108^ ^)^ This delay in the rise of serum RANKL levels supports the possible mechanism of bone loss following denosumab discontinuation, where osteomorphs and osteoclast precursors rapidly form active osteoclasts that resorb bone.^(^ ^17^ ^)^ However, serum levels may not reflect local levels of RANKL; therefore, further study to determine how well serum levels reflect local levels are required.^(^ ^109^ ^)^ Preclinical investigations are pertinent to developing a thorough understanding of the mechanisms driving this rebound bone loss and, importantly, develop definitive sequential therapy approaches to ameliorate the clinical challenges this withdrawal bone loss poses.

With the emergence of the anabolic therapies, such as romosozumab which inhibits sclerostin, complications of discontinuing anti‐resorptive therapy, as well as an aging population and increased prevalence of osteoporosis, attention in the field has turned to how to best utilize these agents over a long period of time for optimal bone mass gain and reduced fracture risk. Despite the decades of research in this field, a “cure” for osteoporosis and other diseases impacted by altered bone resorption still eludes us. As a result, new and future discoveries in osteoclast biology, such as the process of osteoclast recycling, may provide novel avenues to therapeutically target the osteoclast. Some examples of novel osteoclast‐specific therapeutics already in preclinical development include: bone‐specific targeting of the vacuolar H^+^ ATPases of the ruffled border,^(^ ^110^ ^)^ inhibition of the proto‐oncogene tryrosine kinase Src (sarcoma),^(^ ^111^ ^)^ and small molecule inhibitors of adhesion molecules such as osteopontin.^(^ ^112^ ^)^ These are in addition to a number of existing agents or targets in development that target osteoblast–osteoclast coupling factors activin A,^(^ ^113^ ^)^ semaphorins,^(^ ^114^, ^115^ ^)^ and sphingosine‐1‐phosphate.^(^ ^116^ ^)^

## Conclusion

Complex lineage tracing experiments, advances in intravital imaging, and the emergence of single‐cell RNA sequencing have led to paradigm shifts in osteoclast biology, prompting the field to revisit this complex bone cell. These advances challenge historical concepts surrounding the formation, longevity, and fate of osteoclasts, providing new opportunities to further develop osteoclast‐targeted therapeutics. Through these newly discovered abilities to recycle, circulate, and repopulate, the osteoclast population can now be viewed as long‐lived cells with the capacity to regulate more than just degradation of bone matrix and mineral homeostasis. Through the production of coupling factors and direct cell–cell contact osteoclasts can orchestrate new bone formation to maintain skeletal integrity and control elements of systemic energy homeostasis. As we continue to build on this new framework of osteoclast biology with advances in technology, more targeted therapies and treatment strategies will emerge, potentially overcoming the limitations that exist with current anti‐resorptive agents.

## Conflict of Interest

MR received honoraria from Amgen/UCB and Diasorin for lectures. MMM received honoraria from Amgen for lectures.

### PEER REVIEW

The peer review history for this article is available at https://publons.com/publon/10.1002/jbm4.10539.
